# Data as scientific currency: Challenges experienced by researchers with sharing health data in sub-Saharan Africa

**DOI:** 10.1371/journal.pdig.0000635

**Published:** 2024-10-24

**Authors:** Jyothi Chabilall, Qunita Brown, Nezerith Cengiz, Keymanthri Moodley

**Affiliations:** 1 Business Management, Faculty of Medicine and Health Sciences, Stellenbosch University, Cape Town, South Africa; 2 Division of Medical Ethics and Law, Faculty of Medicine and Health Sciences, Stellenbosch University, Cape Town, South Africa; Fundación Progreso y Salud: Junta de Andalucia Consejeria de Salud y Familias Fundacion Progreso y Salud, SPAIN

## Abstract

Innovative information-sharing techniques and rapid access to stored research data as scientific currency have proved highly beneficial in healthcare and health research. Yet, researchers often experience conflict between data sharing to promote health-related scientific knowledge for the common good and their personal academic advancement. There is a scarcity of studies exploring the perspectives of health researchers in sub-Saharan Africa (SSA) regarding the challenges with data sharing in the context of data-intensive research. The study began with a quantitative survey and research, after which the researchers engaged in a qualitative study. This qualitative cross-sectional baseline study reports on the challenges faced by health researchers, in terms of data sharing. In-depth interviews were conducted via Microsoft Teams between July 2022 and April 2023 with 16 health researchers from 16 different countries across SSA. We employed purposive and snowballing sampling techniques to invite participants via email. The recorded interviews were transcribed, coded and analysed thematically using ATLAS.ti. Five recurrent themes and several subthemes emerged related to (1) individual researcher concerns (fears regarding data sharing, publication and manuscript pressure), (2) structural issues impacting data sharing, (3) recognition in academia (scooping of research data, acknowledgement and research incentives) (4) ethical challenges experienced by health researchers in SSA (confidentiality and informed consent, commercialisation and benefit sharing) and (5) legal lacunae (gaps in laws and regulations). Significant discomfort about data sharing exists amongst health researchers in this sample of respondents from SSA, resulting in a reluctance to share data despite acknowledging the scientific benefits of such sharing. This discomfort is related to the lack of adequate guidelines and governance processes in the context of health research collaborations, both locally and internationally. Consequently, concerns about ethical and legal issues are increasing. Resources are needed in SSA to improve the quality, value and veracity of data–as these are ethical imperatives. Strengthening data governance via robust guidelines, legislation and appropriate data sharing agreements will increase trust amongst health researchers and data donors alike.

## Author summary

In our study, we explore the challenges faced by health researchers in sub-Saharan Africa (SSA) with respect to data sharing. While there are many benefits of sharing data, researchers from SSA remain hesitant to share in the context of data-intensive research. Using in-depth research interviews with 16 researchers from diverse countries in SSA, we identified five key barriers to effective data sharing. These barriers include inadequate guidelines and governance processes and fears around ethical and legal issues. If researchers are to reap the rewards of data as scientific currency, resources must be allocated to improve the quality, value and veracity of data. Improving data governance is critical to building trust amongst health researchers and data donors to facilitate the process of data sharing.

## Introduction

Health research and data have evolved into valuable scientific currency that, through technological advancements and cloud computing, present opportunities for researchers to collaborate through sharing data across borders [1]. This discussion of data sharing as currency concurs with Verdegem’s (2021) narrative of AI capitalism [2]. The ‘commodification of data’ [2] has boosted—and even disrupted—research in the fields of science, health, economics and societal establishments. Data sharing has the potential to advance healthcare without the wasteful duplication of repeated research [3–8]. In keeping with the literature [2,8,9], data shared via repositories may be considered as ‘corporate investment(s)’ within ‘merger(s)’ that can amplify yields. As a result, the dominance of artificial intelligence during transformation produces data as tradable capital that can realize substantial scientific gains derived from the reuse, re-analysis and diverse interpretations of empirical data [10]. With proper application of medical data, responsible data sharing through collaborations and on request by external entities, proliferation and publication of research results can be strengthened [2,11–13].

However, data ‘trading’–despite being valuable and increasing researcher capital–must be governed by an organized system to thwart associated challenges [2,9]. Like with any other capital venture, public domains, repositories and other forms of data sharing platforms are not always secure [9]. It is imperative that the sharing takes place within accountable, trustworthy and properly regulated systems promoting the ethical use of data with minimal risk [3,6,7,9,14] with researchers advocating for scrupulous research contexts and reliable reporting [15]. Valid concerns about the associated challenges foster scepticism and prevent researchers from sharing their data on open repositories [9]. Other factors that influence a hesitancy to share data include research acknowledgement negligence, unfair co-authorship, sociocultural factors and ethical and legal barriers [16,17]. The data access and sharing landscape in SSA is distinct from other regions in that it encompasses aspects that are context-specific such as norms, practices and traditions that may obscure international research collaboration [18]. This conflicts with researchers’ moral duty to share data for the advancement and sharing of scientific knowledge [19] for the common good [12,20]. If data sharing in health research is to be trustworthy and properly governed, especially in low-to-middle-income countries (LMICs) it is imperative that researchers are fully aware of the sensitivity of health data in these exchanges [8]. Thus, sensitive health data as well as respondent identities are to be protected to preserve confidentiality.

Such reservations and concerns are not without reason given the many negative experiences faced by vulnerable researchers in, especially, low- to middle-income countries (LMICs) in the region of sub-Saharan Africa (SSA) [6]. Impartial data sharing structures give way to the exploitation and marginalisation of researchers from resource-constrained settings by more influential researchers [12,21–24]. This failure to build research capacity in Africa has been well-documented in the literature [25–29]. Many LMIC researchers are often not acknowledged for their contributions in publications [30–32].This represents a breach of research integrity and is therefore unethical. In certain instances, data has been shared without the permission of authors. When researchers from LMICs are not given opportunities to collaborate with others or when their contributions are not acknowledged it affects research capacity building for the researcher in question. Opportunities to build research capacity through meaningful collaboration and training are missed and this affects researcher output and manuscript quality [33].

Many of the concerns surrounding data sharing play out significantly differently in low-resourced research environments, changing the dynamics of individual researchers’ interactions with Open Data discussions and the development of data sharing practices [18,23,34]. Yet, the interest in data sharing amongst LMIC researchers is ever-growing and researchers in the region recognise the potential of an Open Science future. Stringent governance and management practices are therefore needed to promote secure access to scientific data via legitimate shared platforms as well as to protect the data itself, its contributors and end-users [5,8,12,22]. Although some national and academic institutions across SSA have instituted rigorous data sharing practices, there are still those that rely on what foreign researchers propose [6,35]. Most SSA countries have no regulations in place for the cross-border transfer of data, consequently exposing researchers to untrustworthy practices [7,13,36,37]. Minimal legislation exists across the continent to address the potential implications of data sharing; however, many individual research institutions have taken the initiative to implement their own policies and practices [28]. It is anticipated that African national and regional data sharing policy requirements and the implementation thereof may be expedited by researcher training and government propositions, as has been the case in other countries [28]. Without further empirical research to scope out the extent of interaction, it is unlikely that evidence-based data sharing policies will be developed to truly initiate an Open Data landscape in the Global South [38].

The concept of data sharing must thus be re-evaluated as African researchers themselves become involved with collaborative research and the development of equitable, trustworthy management systems. Benefits for the original researchers must be explicit [6,13,23] as they initiated the knowledge-sharing process [19]. Some studies have found less enthusiasm among SSA researchers to share data where countries do not have enforceable national, institutional or academic policies and guidelines. These researchers are therefore apprehensive and express concerns regarding the misuse of shared data especially since Africa has previously experienced violations in terms of patient privacy [39].

The overall study aimed to establish the perspectives of SSA researchers and research ethics committee (REC) members to contribute to the establishment of guidelines for data sharing in the region. This was initiated in our previous studies [36,37] where we conducted the first empirical survey in SSA in which awareness, perspectives and challenges of researchers and REC members were explored, specifically relating to data-intensive research and data governance. In furthering this aim, we delved deeper into the issues identified through qualitative enquiries and report on our findings in this paper.

Literature reviews revealed a paucity of publications on data sharing legal frameworks and ethics guidelines within SSA. To address this research gap, the following research questions were formulated for the overall study:

- Which frameworks and guidelines are being utilised to guide data sharing in SSA?
- What is the level of awareness of these frameworks and guidelines?
- What legal and ethical challenges exist in health data sharing in SSA?

Additionally, these aims and objectives assisted the researcher in determining the data sharing views and knowledge among SSA health researchers as well as how they mitigate related challenges:

- To identify which countries, have frameworks and guidelines that are drafted, enacted or not yet available.
- To identify the challenges that exist in countries with and without data sharing frameworks and to brainstorm potential solutions.
- To explore previous data sharing experiences of health researchers.

This paper explores the challenges health researchers experience in data sharing in SSA. The qualitative enquiry is a baseline study to capture a sample of diverse continental views in SSA.

We first illustrate the methodology, in terms of the assumptions underlying the study design and implementation, the study design and sampling, data collection and analysis and ethical aspects. This section is followed by the results that elucidate themes and sub-themes generated after in-depth analysis. The final sections are the discussion, theoretical and practical contribution and implications, limitations and finally, concluding thoughts.

## Methodology

### Assumptions underlying the study design and implementation

For the purposes of this publication, we clarify that researchers invest time, funding and energy to create a comprehensive research project referred to as the capital. To stimulate rigorous health research, it is imperative that researchers can network confidently within a controlled data sharing sphere fostering mutual feedback and acknowledgements [23].

The qualitative enquiry elucidated the opinions and experiences of SSA researchers in terms of data sharing. Participants’ views advanced the researchers’ understanding of the benefits of data- and knowledge-sharing, recommendations as well as ways to mitigate related challenges. In their efforts to brainstorm potential answers [40], the research team has worked towards identifying countries that have drafted or enacted frameworks and guidelines and those that do not have these governance facets. Researchers who participated in the study under discussion accepted the premise that where there may be inadequate data sharing regulations, there is a dire need to create transparent obligatory conditions in each SSA country. It may be noted that a uniform set of guidelines may not accommodate all countries in this region adequately.

### Study design and sampling

A qualitative study design was used in which we explored the views of health researchers in SSA on data sharing and the related challenges and opportunities. In-depth interviews were conducted with this baseline cohort from 16 different SSA countries. Thirteen of those countries were Anglophone given that the research team were English-speaking. In three Francophone countries, the respondents were fluent in English. The 16 countries included the five most research-intensive countries in SSA in the fields of public health, environmental and occupational health–South Africa, Nigeria, Kenya, Uganda and Ethiopia [41].

The sample was recruited through a purposive selection of professional networks of the Division of Medical Ethics and Law and the School for Data Science and Computational Thinking at Stellenbosch University. As many researchers as possible who were involved in data-intensive health research were invited to participate in the study. The selection criteria included researchers working within the health or public health sector, being 18 years of age, living and working in an SSA country, and being able to provide consent. The initial search for participants was broad and intended to reach as many SSA countries as possible. We used multiple sources to identify and recruit initial contacts, such as online platforms. Where publicly available, we made use of university and research institutions’ websites to identify and invite (via email) potential participants based on background and job title. Only 16 health researchers were available to participate in in-depth interviews.

Unfortunately, the response rate was sub-optimal. This is a common challenge in conducting empirical research in SSA [18,34]. Due to the relatively small proportion of health researchers in SSA who play multiple roles in teaching, research and health service, it is often challenging to engage participants in empirical qualitative research mainly due to their health service obligations in severely under resourced settings on the continent. During initial recruitment, the response rate was low due to challenges such as poor internet connectivity, power interruptions and limited email access. etc. To mitigate these challenges, snowball sampling was also introduced for further recruitment. Hence, at the end of every interview, the interviewer would ask participants if they knew other health researchers in other SSA countries who would fit the study criteria and who would like to participate. A combination of both sampling techniques was used. Sampling was purposive in that we tried to include different SSA countries to obtain a broad overview of perspectives in the region. We were also looking for health researchers involved in data-intensive research. Here snowball sampling was useful due to large collaborative research projects in SSA funded by EDCTP, NIH and Welcome Trust. Overall, all attempts were made to mitigate sampling bias by contacting researchers from academic institutions where most research is conducted in Africa as well as funded projects.

This study is linked to a previous descriptive cross-sectional online survey in which we invited respondents to anonymously participate through Research Electronic Data Capture (REDCap) [37]. At the end of the questionnaire, respondents were asked to indicate their interest in participating in an in-depth interview. For those who agreed and provided their email address (through anonymised branching logic which directed them to a Google Form), consent forms were sent directly to them. These in-depth interviews are intended to provide a broad context on the issues identified in the questionnaire [37].

### Data collection

Although invitations to participate were sent to many scientific researchers across SSA and were requested to invite colleagues to participate within their own personal capacity, we were unable to obtain many positive responses. Other studies conducted by the group also demonstrated the time- and work-related challenges that African researchers encounter–preventing them from participating in such studies. JC and NC contacted the researchers to schedule a date and time that would be convenient for them to participate in the study. After obtaining initial consent, 16 in-depth interviews were conducted via Microsoft Teams. Data collection commenced on the 28 July 2022 and ended on the 17 April 2023. Interviews took place at the workplace or the home of the respondents. All interviews were conducted in English and lasted an average of 45 minutes. Informed consent forms were sent to respondents prior to the date of the interview, and they were encouraged to ask any questions related to the study. Consent was then recorded on the form and sent back via email to JC and NC. Before each interview, JC and NC reiterated details of the study and confirmed consent with each respondent. In these discussions, respondents verbally agreed to be recorded in each interview.

The semi-structured interview guide was based on a literature review and expert consultation with colleagues from the School for Data Science and Computational Thinking, Stellenbosch University. The interview guide covered the following aspects: participants’ background and function at their institution, their understanding and experiences of data sharing, views on challenges to data sharing, knowledge of existing guidelines to data sharing and perspectives on the development of an inclusive data sharing guideline policy (refer to the interview guide under S1 File).

Countries represented in the sample include Botswana, Burundi, Democratic of Congo (DRC), Ethiopia, Eswatini, Ghana, Kenya, Malawi, Mauritius, Namibia, Nigeria, South Africa, Tanzania, Uganda, Zambia and Zimbabwe. Thirteen of these countries are considered Anglophone, while the remaining three are Francophone. Table 1 refers to the researchers’ professional roles and the type of research they are engaged with. Most researchers are currently employed in the public health sector and serve dual roles (teaching or practising medicine).

**Table 1:** Breakdown of respondents.

Participant Number	Role	Type of Research
IDI 1	Researcher	Public Health
IDI 2	Computer Scientist, Researcher, Lecturer	Public Health
IDI 3	Researcher	Geospatial and Public Health
IDI 4	Researcher	Public Health
IDI 5	Researcher, Lecturer	Public Health
IDI 6	Medical doctor, Researcher	Public Health
IDI 7	Researcher	Public Health
IDI 8	Lawyer, Researcher	Public Health
IDI 9	Researcher, Lecturer	Public Health
IDI 10	Bioinformatician, Researcher	Public Health
IDI 11	Researcher, Lecturer	Public Health
IDI 12	Researcher	Public Health
IDI 13	Medical doctor, Researcher	Public Health and Policy
IDI 14	Medical Virologist, Researcher	Public Health
IDI 15	Researcher	Public Health
IDI 16	Pharmacokinetics, Researcher, Lecturer	Public Health

### Data analysis

Audio files were cleaned, transcribed verbatim, and thematically coded using ATLAS.ti (version 22). Each interview was coded independently by QB using a combination of inductive and deductive reasoning for theme development. While the deductive analysis traced the themes listed in the interview guide, the inductive analysis allowed the expansion of the list of themes, by adding those that emerged from coding the interview content.

Intercoder reliability (ICR) was established whereby SK independently coded a section of the dataset. O’Connor and Joffe recommend that when evaluating ICR, a small subset of the dataset should be coded and 10–25% of the data units would be sufficient [42]. This translated to SK coding 3 transcripts which amounted to 20% (3.2) which were randomly selected. Establishing ICR was important as it promoted the transparency of the coding process and stimulated discussions between SK and QB in relation to the qualitative themes that were developed [42]. The data analysis was performed by QB and was based on the thematic method proposed by Braun and Clarke [43]. Major themes of interest were identified and categorised, followed by an in-depth analysis of the themes through discussion among the team of involved researchers. Adjustments to the final thematic map were made to improve logical cohesion.

### Ethical aspects

Participation in this study was voluntary. The in-depth interviews posed a minimal risk as the sample included educated and empowered respondents who had full capacity to consent or decline participation. We approached our respondents in their capacity and respondents consented in their personal capacity. Ethics approval was granted by the Faculty of Medicine and Health Sciences Health REC (Reference No: N22/03/028) at Stellenbosch University, South Africa. An application to extend ethics approval was made and granted for the period of 19 April 2023 to 18 April 2024.

## Results

Qualitative themes that were acknowledged comprised participants’ current data use and access patterns, beliefs and practices in data sharing and opportunities to promote data sharing in SSA. The latter findings will be reported on in a companion paper. This paper focuses on the findings that emerged regarding the challenges and barriers to data sharing that researchers currently face [16]. Themes include individual researcher concerns, structural factors impacting negatively on data sharing, recognition of research in academia, ethico-legal challenges to data sharing and lack of regulatory frameworks [17]. Several subthemes were developed, and this is discussed in relevant sections.

### 1. Individual health researcher concerns

This theme focused on individual-level factors that act as barriers to data sharing within the research process. Two subthemes are presented, namely, fear regarding data sharing and publication and manuscript pressure.

#### 1a) Fear regarding data sharing

Respondents in this study expressed reluctance and fear regarding sharing of data in the context of collaborative research even on secure repositories for various reasons. Researchers from low-income countries were particularly concerned that sharing their data might lead to other more resourced researchers stealing their ideas. Hoarding data and research often occur as a result of these fears because researchers have experienced some type of misuse of their data and work. These sentiments were expressed by respondent IDI 11 below.

*“it appears that most scientists are not comfortable in bringing their data into one repository”* (IDI 11).

*“people sometimes hoard their data because they try and develop ideas and exhaust their ideas because some people run with your ideas”* (IDI 11).

*“There could be more benefits because many people are scared of actually sharing the data*. *Given… all the issues that come up*, *you know in terms of ethics*. *So*, *people are quite worried unless you have the framework*, *you know*, *a proper secure medium…”* (IDI 7).

Respondents also indicated that there was a sense of unwillingness among some researchers and stakeholders to share data. This leads to frustration for researchers because even though funders require data sharing, this often does not materialise and could impact the timely submission of publications.

*“… even as part of the NIH data policy participating members of the consortium must be willing to share that data. It was only two people that were willing to share their data”* (IDI 1).

*“NIH demands that researchers indicate that they’re going to share data…*, *that’s what is happening here in [my country]*. *They accept that they will share their data but…that data sharing is not happening*.*”* (IDI 11). A few participants mentioned data sharing as a publication requirement. This was mostly acceptable with certain safeguards. A view was expressed as follows:

***“****Actually*, *many of the journals these days insist that if they are going to publish your manuscript*, *then you should provide that data*. *It’s increasingly becoming a norm from the journals that data is shared*, *before*, *when they accept your manuscript then they ask you to deposit the data either in a public repository where it can be accessed or as an attachment to your manuscript*. *I’ve done it and I think it’s going to continue*. *In the past*, *it wasn’t much stress but these days since its increasingly a requirement*, *I think we should be able to do it… if you’re going to publish a manuscript*, *there is a requirement by some journals that you sub- share data and the idea here is you can share data that is related only to that manuscript*. *In that way you are sort of protected on how the data is shared”* (IDI 9).

Respondent 9 further expressed that sharing data within the context of journal publications can be challenging as it raises concerns about how to ensure the anonymity of the data.

*“The only way is how do you make sure you adequately anonymize that data so that it doesn’t pick out the individuals or the community that was affected and how do we prevent that data from being used by individuals who may not really uphold the ethical practice? In the end the data becomes a danger to the community where the research was done”* (IDI 9).

#### 1b) Publication and manuscript pressure

*“… being in academia, you know, research, they say you either publish or you perish”* (IDI 2).

A subtheme that developed was the institutional and peer pressure upon researchers to publish and accelerate manuscript writing. Moreover, a lack of support systems at academic institutions and an inability to find innovative ways to increase equitable sharing amongst researchers were highlighted as factors that hinder data sharing and academic publications.

*“The problem has been the fact that we have not been able to devise strategies for understanding win-win approaches in data sharing”* (IDI 1).

*“… we may not have systems in our institutions to help us really analyse the data fast*, *develop manuscripts*, *and then draft them as fast as possible”* (IDI 9).

Publication pressure can be viewed as having a potentially damaging effect on the individual researcher during their academic career. This negative effect was underscored by respondent IDI 9, who indicated that during certain times such as pandemics, publication pressure can result in researchers falsifying and fabricating data to advance their careers. In this case, important decisions were made based on erroneous information that could have dire societal and health consequences.

*“…It’s very helpful if you look back sometime during the peak of the COVID-19 outbreak, individuals were publishing information that was affecting policy and decisions based on non-existent research. It was fabricated… it was found out later when they were asked for databases or data sets that they had never conducted that research, but then their publications they are already negatively affected some”* (IDI 9).

In some instances, publication pressure is exacerbated by unreasonable funder requirements such as the time allocation to publish. Respondents stated that often the time is insufficient to capitalise on their data and increase their publications. Respondent IDI 12 stated that funders should be cognizant of this and acknowledge the effort that goes into preparing a specific dataset. This awareness could serve two purposes, firstly, alleviating the fears that researchers have regarding data sharing and secondly, reducing the duplication and potential loss of relevance of research studies.

*“…I know some donor-funded data that will come with those requirements that you will only have a year once you have completed your study to finish up your publication and then make the data public… so that other researchers can access it. Often than not I find that the period sometimes is too short for researchers to complete that really good analysis and come up with the write-ups before you know it, you wanted to really continue to have some writeups as a researcher, but now we are forced to put the data out there for other people to publish, yet you’ve invested so much time, you know… collecting the data, analysing the data, and then you only publish one paper or two… Again, researchers should not hold the data for themselves for long until it’s… no longer relevant”* (IDI 12).

### 2: Structural factors impacting negatively on data sharing

Structural constraints mentioned by respondents included poor resources, lack of proper data infrastructure and data management. An absence of adequate technological systems impacts the ability of researchers in SSA to digitise their work. This constraint, coupled with expensive forms of data storage such as cloud access, means that researchers are unable to practice good data governance which in turn leads to loss of valuable data.

In terms of structural factors, respondents expressed the following concerns:

*“…so, I think it’s data availability, data storage, data processing, computer illiteracy, digitalisation problems”* (IDI 5).

*“… if you don’t have resources to put your data to the next level of the value chain*, *you’ll be the last to benefit from your data”* (IDI 14).

*“… but when it comes to the context of the resource constraint*, *overworked researchers in Africa…want to generate insights from the data but sometimes it goes beyond the timeline that is provided by the funders*, *and they’re not able to do that”* (IDI 1).

*“… cloud access is a bit tricky … the cost of the … cloud can*, *can go up very high if you don’t know how to manage that well … if people don’t know how to switch off the VM and how to use that*, *cloud access can be very expensive”* (IDI 7).

*“Many times*, *data gets lost*, *and the gadgets get corrupted where we store the data”* (IDI 9).

*“… think about the amount of time and effort … to prepare this data for sharing they actually give up because they don’t have the time…*. *But if they had managed their data well from the very beginning*, *then when you tell them*, *can you share your data*, *it’s almost like clicking a button”* (IDI 10).

### 3: Recognition of research in academia

The nature of science, particularly the way academia is presently structured was highlighted as a challenging factor related to data sharing. Two subthemes emerged as noteworthy barriers [16] to data sharing: the scooping of research data and the role of recognition or acknowledgement and incentives within academia. These are discussed below.

#### 3a) Scooping of research data

Concerns were raised around the concept of scooping, the fast pace of research, competitiveness, the confidentiality of science and certain scientific fields gaining prominence over others (for example epidemiology). Scooping has been defined as “a research community slang term, for having someone else claim priority, usually through publishing, to a research idea or result you yourself have been working on” [44]. Funding availability and regulations were expressed by researchers as severely limiting their efforts to data sharing and advancing in their respective careers.

“*The concepts of scooping and all those people where if they see your data there and then you are addressing exactly the same question… only they are able to analyze the data faster… so… African researchers have not seen a lot of benefits that is in data sharing”* (IDI 10).

*“…and you know*, *there are no regulations to support*, *to promote researchers coming up with… funding to share the data as well”* (IDI 1).

#### 3b) The role of recognition/ acknowledgement and incentives in research

For researchers working in the SSA region, being recognised for your research and receiving acknowledgement and incentives for data sharing is extremely important. Unfortunately, many researchers indicated that in many instances they had not been acknowledged or knew colleagues who were not recognised for their research endeavours.

*“And also, there’s some of the conferences that I have attended. I was in the Health Systems Global Conference in Cape Town, and they were facilitating the data on the community initiatives in Malawi, in Uganda and I didn’t see the researchers in those countries acknowledged in those presentations…”* (IDI 5).

Lack of acknowledgement of researchers and communities was underscored as troubling and contributes to fears of sharing and mistrust of others, especially amongst low-income country researchers. Often researchers in collaboration with research participants are viewed as merely data generators who are not given the opportunity to contribute to publications. This affects researcher growth and the career trajectory of the researcher.

*“… data is … a scientific currency, that is what the researchers use to generate the output that has incentives… publications… that I use for promotions… and just recognition even within the field”* (IDI 10).

*“We have seen it during the COVID pandemic that the people who generated all the sequences that were used to generate new diagnostics*, *new keys*, *new vaccines*, *there was total disregard of people that generated the data and that’s very sad”* (IDI 14).

*“There’s a bit of not all parties benefiting… we’ve been affected by what they call helicopter research whereby*, *African researchers and scientists are only used to collect data or to collect samples*, *right and then the samples and everything is taken to the West … and the data analysis is done there and not much recognition is given to the data they give you”* (IDI 10).

### 4: Ethical challenges related to data sharing

Ethical concerns were recognized as a contributing factor impeding data sharing amongst researchers. Ensuring research integrity by adhering to research principles were highlighted as potential challenges such as confidentiality and informed consent. This subtheme is discussed below.

#### 4a) Impact of data sharing on confidentiality and informed consent

Respondents’ concerns regarding confidentiality were centred around questions of sharing with others and risk. Others highlighted the importance of ensuring informed consent from the outset to reduce potential ethical challenges.

*“Because we’re saying that we want to keep this one as confidential as possible. But the other side is, how confidential and how secure is it when we begin to share with two, three people or more. But it’s not guaranteed so that risk is always there… we turn a blind eye to it because it seems like we don’t have a solution right?”* (IDI 2).

*“… Respondents were not aware whether their data will be shared*. *Of course*, *the minimum ethical requirement is to de-identify all those data*. *To de-identify the population but the question of the ethics got me*. *Now if such information was not requested from respondents at the beginning*, *it becomes a problem*. *Who decides -is it the researcher or the REC*? *What will be the role of the REC in this situation*? *So*, *it’s*, *a conundrum of several factors that have not been actually figured out”* (IDI 1).

The role of RECs and ethics oversight were underscored, and researchers questioned whether their RECs were capable of mediating some of their perceived concerns. These are pertinent concerns that necessitate the need for proper discussion between all the essential stakeholders to ensure that RECs are appropriately capacitated to address and resolve all ethical issues.

*“No, nothing in law yet, but the REC, they try to ensure that in your protocol. Tell us how you disseminate your data… what would be the benefit, you know, to the population at large. So, researchers would describe all of that, but no one will, at the end of the day, put you to task if you have not done that”* (IDI 12).

Interestingly, respondent IDI 1 mentioned below, that confidentiality within science can also serve as a barrier to data sharing amongst researchers in the Global North and South. This is often the case when it comes to groundbreaking research which is rarely shared with researchers from other countries.

*“it has to do with the confidentiality of the science. When they talk about the diversity in Africa… for my counterparts in the US and other high-income countries. So why not share your own data? So, for example, if you heard of the Framingham Heart Study in the US, nobody has access to that data except researchers in the US… So why is it being kept—it’s about the confidentiality of science”* (IDI 1).

#### 4b) Commercialisation and benefit sharing

The commercialisation of data was expressed as a factor that impacts researchers’ data sharing, particularly in the context of research participants and benefit sharing. Below, respondent IDI nine has expressed that commercialisation especially of genomic data has the potential to become problematic as this involves many complexities such as consent and ownership of data.

*“The other challenge we get with sharing data is for example, companies or individuals that are interested in commercializing some of this data, like genomic data, they might pick that data and use it to develop commercial products or to verify or, to use them to standardize whatever they are testing and then develop products. The question is if you’re going to use that data to develop a commercial product, did the participants give consent for that?”* (IDI 9).

Respondent IDI 16 further emphasizes that clinical trials and the role of pharmaceutical companies is a contentious issue and this needs to be debated given the potential of publication bias and the multiple ethical challenges (such as conflict of interest) that this could pose.

*“…randomised clinical trials, it’s something that obviously I follow because it’s very interesting. It’s very important for drugs to be marketed on the basis of very good trials. And then I know obviously that if you look at clinical trials uh, if they are sponsored by the pharmaceutical industry, there is a big problem, and even when they are sponsored or, carried out by public type institutions the problem is publication bias”* (IDI 16).

The relationship between the commercialisation of data and benefit sharing was articulated by respondent IDI 15 as a major ethical issue. This is largely due to concerns related to how communities’ benefit, either directly or indirectly and needs to be acknowledged, discussed and managed. This is further complicated by funders or donors who are proponents of open science, yet they have failed to take into consideration if and how research participants and their communities are benefitting from the data that they generate.

*“Now, I had never envisaged that in my ethics that it is possible that somewhere down the line, other researchers using this data may have some positive advancements from a commercial standpoint. Where does that put this community? Because I would say, well, whatever I’m doing, if there’s anything I promise to declare it to you, and if there’s something that is of monetary value, we will share that. You can set up all these a priori, as a primary researcher. Secondarily, down the line and it brings me back to the starting point about the communities that contribute to participate. What benefits do they get and how? How do they advance themselves in the local setting?”* (IDI 15).

*“I know some funders are pushing for open and data access*, *but sometimes it’s also harmful if you don’t look at the needs of the people who generated the data*, *who sometimes may generate data about for instance*, *a vaccine*, *but they may never have access to the vaccine… Sometimes*, *you know that you have no direct benefit*, *but I think in the spirit of benefit sharing that often gets overlooked when it comes to open access*, *open sharing of data”* (IDI 14).

### 5: Legal challenges: Lack of regulatory frameworks

The lack of proper regulatory frameworks which respondents perceived as leaving them without legal protection was recognised as a potential barrier to data sharing amongst researchers.

*“… there was no law or there was no push to say can you please share the data as soon as you finish… because there was nothing to push people to share data so everyone was doing what they felt like and at their own pace”* (IDI 11).

In certain SSA regions, data sharing is not prioritised at the governmental level, and this impacts researchers’ capacity and ability to share data and progress in their academic careers. Countries such as Ethiopia and the DRC currently do not have comprehensive data protection laws in place [45].

For researchers who have some form of framework or standard operating procedures (SOP), this is not legally binding. Respondents indicated that the absence of frameworks and legislation hinders data sharing for SSA researchers.

*“… these are SOPs which are not necessarily protected by legal… or policy at the moment. So, these are just at SOP level which when someone maybe is not adhering to, we still don’t have the legal consequences as yet as a country”* (IDI 12).

Respondent IDI 12 further stated that the absence of relevant data protection laws can also be the result of a political lack of will and poor resources needed to draft and promulgate laws in their country. This is further exacerbated by a lack of research coordination at the governmental level and a low level of capacity needed to strengthen data sharing in countries where there is limited legal protection. Respondent IDI 8 expressed similar views. Both respondents are from countries that do not have sufficient data protection laws.

*“It will also require a bit of influence, political will and resources as well but I think I’ll speak to our context of [my country] again, when within the government, we know that there’s a research department, there is a health department within that research department. I think one of their objectives is to coordinate research, knowledge translation but often… there is no capacity*” (IDI 12).

*“But when it comes to policymakers*, *I’m not sure they’re aware of the issue…for countries like us where there are other pressing issues this kind of issue may be considered as a luxury and so…I’m not sure if our policymakers or regulatory bodies would be interested in this area*, *but somehow as a responsible citizen this is something we have to address sooner or later”* (IDI 8).

## Discussion

In the era of big data and artificial intelligence in healthcare, data sharing linked to publication and during collaborative research is lauded as an important scientific practice to enhance health benefits, streamline workflows in health systems and stimulate innovative health interventions. These positive outcomes of data sharing are widely embraced yet significant challenges exist to escalate data sharing. This qualitative study highlights some of these challenges. In particular, an important concept of researcher vulnerability with respect to data sharing amongst respondents from a sample of SSA countries is illustrated. Health researchers expressed personal fears and professional reluctance to share data. In addition, concerns were expressed about data quality due to resource limitations and structural constraints to data capture, curation, storage and sharing. Several ethico-legal concerns were also raised. These interrelated factors play a contributing role in researchers’ decisions to share their data against a background of suboptimal governance frameworks to protect data and prevent exploitation in the context of collaborative research in SSA. While the challenges in data sharing have been presented as separate themes, it is best to interpret the challenges as interrelated, where one barrier has a cumulative effect on the next one identified by researchers, given the complexity of data sharing in health research in low resource settings. This is consistent with the approach of Van Panhuis et al. (2014) where the authors presented barriers to data sharing within the public health sector and emphasised that barriers are interrelated and should be studied and addressed holistically [46]. Challenges at the individual level such as fears regarding data sharing and collective structural factors such as poor resources and inadequate technological systems, ecosystem level factors at the institutional level (in this case, the way recognition and academia are structured) and macro level factors (ethical and legal challenges) all interact with each other and these barriers collectively serve as challenges to data sharing for SSA researchers and can impact on researcher growth and career progression.

Researcher vulnerability in low-resource settings has been exacerbated in the context of data intensive research. While researchers in resource-rich countries also experience conflict in terms of the need to be recognised as reputable researchers [47], academic pressure to publish and a protectionist attitude toward their research data [48,49] this conflict is more acute in LMICs due to the asymmetrical power relationships with international collaborators in HICs and funders from HICs [9,25]. More specifically, researchers in LMICs fear exploitation by international partners [50] and local competitors alike [51]. These fears have been previously highlighted as a factor that impedes data sharing and are largely attributed to the past misuse of data and research by HIC researchers primarily towards LMIC researchers [30,31,52]. Crewe et al. (2020) found that this fear varied depending on the age of the researchers in their study [53]. Likewise, Hodonu-Wusu et al. (2020) surveyed a sample of 135 researchers from Malaysia who disclosed fears of the potential misuse of data and lost opportunities to publish impacted their data sharing endeavours [54]. Similar findings were reported in a study of Spanish researchers [55]. Researchers in our study spoke about their frustrations in relation to lack of acknowledgement and recognition for collecting the data and preparing the datasets. This finding is consistent with research findings discussed by Alter and Vardigan (2015) in their review of data sharing attitudes of researchers from five countries (Kenya, India, Vietnam, South Africa and Thailand) [30]. Compounding fears of exploitation amongst researchers in SSA is the fact that academia is a highly competitive environment, and many researchers experience pressure to advance by increasing their publication records [56]. Literature has demonstrated that for researchers in LMICs, this appears to be even more daunting as they are faced with challenges such as limited time to prepare and write up their findings for publication [38]. In India, an exploratory study revealed that publication pressure is felt more intensely amongst female researchers, early-stage researchers and researchers working in the fields of social sciences and humanities [57]. Our study shows this to be an important concern amongst health researchers as well [58]. The fear of being scooped by other researchers was a major impediment to research career advancement and the promotion of data sharing for SSA researchers. This finding is consistent with empirical evidence found across different disciplines and contexts [47,59,60]. Interestingly, researchers in our study underlined that some research fields such as epidemiology receive more recognition for data sharing, which appears to limit the potential for career advancement in other fields where data sharing is not frequently practiced. This is not a surprising finding given that research has shown that data sharing within certain disciplines such as in the natural, technological and biomedical sciences is more common than in the social sciences and humanities fields [61]. Funders could play a considerable role by easing strict publication requirements and by promoting data sharing amongst scientific fields that are generally challenged with sharing their work with others [15]. Sharing data with journals within the context of publication elicited fewer concerns. However, researchers highlighted the importance of only sharing data related to a specific publication and not all data related to the study. This way they would be able to use their data sets for additional publications. The challenge of anonymizing data to share with journals could be addressed in research budgets so resources are available to anonymise data for sharing.

Another significant concern is that of data quality in LMICs. Beyond individual concerns, there are broader, comprehensive barriers to data sharing that prevent the seamless exchange of research information. This has been widely documented in the literature as playing a role in limiting data sharing, especially in resource poor LMICs [32,38,62,63]. Researchers in our study confirmed that structural barriers such as lack of resources, inadequate technological systems, suboptimal funding and lack of electronic health records make it challenging to capture, curate, store and share data. This is consistent with findings from Rappert and Bezuidenhout (2016) who reported that in their review of two sites—Kenya and South Africa- structural barriers such as inadequate technical support and repeated power cuts are challenging to their everyday research endeavours which impacts on data quality, data capture and data sharing. Health researchers in SSA play multiple roles in health service delivery and teaching, with proportionally less time for research [64]. Dedicated research time is also important to improve data quality. As with all types of research, scientific integrity of data is a foundational requirement. It is also a profound ethical requirement [65].

The global stimulus for data sharing acknowledges that the veracity of data is equal around the world. However, health researchers in LMICs including those in SSA express concerns over funding for the collection, cleaning, processing and storage of data to ensure high-quality data that is suitable for sharing [37]. The implications for health research funding are therefore profound. For researchers working in SSA and collaborating and competing with better resourced colleagues it is imperative to strive towards a more egalitarian approach [50] which is an enormous endeavour requiring the commitment of many stakeholders. Many of these challenges are less acute in HICs where research is well-funded and data management is well-resourced. Improving data sharing in LMICs requires systemic change that entails a concerted effort by funders and all stakeholders involved when developing partnerships and assigning budgets [66]. This would go a long way in promoting data sharing on the continent. Buckee et al. (2022) established post-covid insights from novel data streams that could help drive precise, impactful health programs, and bring effective aid to communities [67]. Researcher participants were generally in agreement that ethical challenges such as deficient or incorrectly formulated informed consent procedures and the lack of confidentiality are commonplace within the context of research. Given suboptimal data literacy in SSA, consent processes in data intensive research are at risk [37]. This finding was also highlighted by the work of Kabanda et al. in the online survey exploring data sharing among researchers from SSA [37]. Relatedly, in that sample of SSA researchers, most of the respondents interviewed were from the public health sector working with sensitive health data that requires extra layers of protection to ensure confidentiality and anonymity as well as informed researcher decision-making. Hence, it is not surprising that these ethical concerns were raised [37]. The importance of adhering to ethical principles has been raised in both international [68] and local contexts [13,30] and is regarded as essential in attaining the goal of ethical data sharing practice. To provide solutions to the issue of consent, several authors have argued that consent must be re-examined to include options such as broad and tiered consent models especially in the genetic and genomic research landscape [69,70]. Improving data literacy is critical to enhance consent processes in LMICs. Unlike HICs where data literacy is potentially higher, the risk to consent processes is likely to be a less serious challenge.

Data governance emerged as another important challenge to data sharing amongst our respondents. Some countries in SSA have data protection legislation, but researcher awareness thereof and the extent to which these regulations are accurately implemented is doubtful [34,71]. In the South African research arena, the Protection of Personal Information Act (POPIA) seeks to control the irregular sale of data to safeguard researcher interests [72,73] giving precedence to rigorous data sharing governance [72]. Other countries like Kenya, Ghana and Nigeria have their own data protection laws [74].

The European Union’s General Data Protection Regulation (EU-GDPR) usually impacts collaborative research projects with Europe [75]. In China, Europe and the USA, extensive legislation has been developed regarding data protection i.e. EU-GDPR, EU-AI Act and HIPAA [76]. While there are efforts to develop a comprehensive form of protection for the African Union (AU) this is still underway leaving many countries without protection [77].

Respondents spoke at large about benefit sharing [78] and what can be deemed as equitable. These discussions were mainly focused on the potential benefits for research participants and their communities. The concept of benefit sharing is complex where indigenous communities often express a strong desire for equitable distribution of the benefits of research to enrich the standard of wellbeing during proficient healthcare [79,80]. Various ethics scholars have advocated for benefit sharing as an altruistic endeavour underpinning friendliness, fairness and reciprocity [81–83]. More specifically, Munung and de Vries (2020) have argued that “the idea that study communities had provided valuable resources (samples and data) for research purposes and by extension, for the advancement of science and thus fairness required that they receive some sort of benefits” [81]. Moodley et al. share similar sentiments around fairness and justice as those who bear the burdens of research should benefit [69,70,84]. The HUGO Ethics Committee proposes that companies engaged in health research offer 1–3% of net profits to healthcare infrastructure or humanitarian efforts as the genome is recognised as a common resource [15,69].

The lack of regulatory frameworks was highlighted by researchers as limiting efforts at data sharing in SSA. More specifically, respondents found that there was a dire need for more structured data sharing guidelines and administrative systems that would promote confident honest data sharing practices. This is similar to other studies in SSA [11,32,51,84]. Researchers in SSA acknowledge that it is acceptable that researchers across the world participate regularly in similar research projects and are willing to share their data albeit under the terms of sound regulations and data sharing agreements [11,85]. Although they may not have access to the necessary management regulations, African researchers are fully aware that they ought not to be uploading data onto repositories that do not insist upon adherence to access regulations.

While it is crucial that only high-quality data is shared on trustworthy platforms, there is also the need for that same scientific currency to allow for quality expansion, re-interpretation and re-assessment. At the same time, acknowledgement of the original data researchers’ capital will require trustworthy data sharing within a regulated environment.

Expeditious access to high-quality scientific currency can flourish into innovative follow-up findings, benefitting both the initial donor of the data and the subsequent consumer. Researchers risk losing their standing as credible researchers should they merely seek benefits rather than honest sustainability of the capital, via data re-use and re-interpretation for the advancement of science. With regards to promoting data sharing amongst SSA researchers, the *FAIRER* model [86,87] is becoming widely adopted [12,17,88]. The guiding principles of this model propose that data ought to be findable, accessible, interoperable and reusable [12]. In the interests of democratic scientific advancement and integrity within the data sharing context, the World Health Organization [89] and other academics [12,17] endorse the *FAIR* principles [86,87]. Data scientists are encouraged to contribute quality data to trustworthy well-managed data repositories especially if there are complementary reward systems [90]. At the same time, secondary researchers will find quality data on the same repositories that are well-described, structured and anonymised [17]. Our findings echo the results of other studies that call for *cutting-edge FAIRness*” [86] and far-reaching foundational support if there is to be the protection of data as a valuable currency available on shared platforms [7]. The lack of this fundamental local reinforcement can lead to unrealistic claims of data and asymmetrical power relationships in international research collaborations that are widely documented in the literature [38,53].

Rigorous transparent, ethical and legal regulations governing data sharing processes are essential to guarantee researcher integrity and responsibility and to prevent the re-interpretation of superfluous data [8]. There is a proliferation of innovative strategies once data donors authorize other researchers to upload current trustworthy data via data sharing platforms. While stimulating novel research questions, methodologies, and sources, and reducing the inconvenience of reiterations, governance of intellectual property regulations [91] can also prevent hegemonic practices sometimes displayed by unethical researchers [8].

## Theoretical and practical contribution and implications

Data is perceived as scientific currency that is shared to provide researchers with the opportunity to accelerate their careers and publications often serving as the beneficial [9] basis for promotion and future funding opportunities [13]. However, within our sample of SSA researchers, data sharing in the context of collaborative research is perceived in a negative light largely due to past experiences of not being recognised or acknowledged or being inadequately incentivised for their data and work. This finding is not new [40,51,59] and speaks to the urgent need to tackle this serious concern to enhance the benefits of data sharing in the African context. Anger et al. (2022) have argued for the need to renew how researchers should be rewarded for their data and their efforts to share data with others [1]. Funding agencies are central to this reform [9], as they emphasise evaluation metrics that encourage data sharing by underscoring other valuable ways of contributing to scientific outputs, such as the collection and preparation of datasets [1]. Ross et al. (2023) and Perrier (2020) states that ‘(a)lthough funding agencies, institutions, and journals have implemented policies on data sharing and archiving, these practices have not produced the anticipated results.’ [9,91]. The ideal, according to Ross et al. (2023) is that, as an example, the NIH could create a central platform for widespread use [9]. Such publicly funded innovations will provide the necessary support and facilitate large-scale data storage and more stringent governance and security measures [80,92].

Similarly, Anane-Sarpong et al. (2020) have provided a comparable argument by calling on the scientific community to develop “data-sharing rewards that have comparative professional weights to the current ultimate of authorship; or make quality data production a sufficient criterion for it” (p.90) [38]. Moreover, Westoby et al. (2021) have advocated for including another type of currency for researchers to progress and expand their scientific contributions aptly named “data contributions” currency [12]. To this end, they have developed a model documenting the advantages of using a data contribution statement to improve the practice of data sharing by providing researchers who primarily work with data to reap the rewards of their work [12].

The commercialisation of research data was a prominent topic among researchers. Researchers spoke of the complexities of commercialisation and how this practice has ethical implications. This is especially pertinent when it comes to genomic data–as our respondents confirmed. These concerns have been documented in the literature [69,70,93]. Findings illustrated by respondents concentrated on informed consent and how it impacts commercialisation and benefit sharing. Without a doubt, commercialisation remains a contentious topic that requires extensive debate and discussions with all relevant stakeholders. The commercialisation of data in the South African healthcare and health research context is intricately related to issues of informed consent, access to and the sharing of data, as well as privacy and the confidentiality of personal information [8,71,91]. Commercialization of data ought to be sustainable and preserve mainly the quality of the data [8].

Finally, data quality remains a major threat to data sharing. More consideration of data management costs is required by researchers and funders alike to ensure high-quality, reliable datasets are made available for sharing [15]. Health researchers in SSA play multiple roles in health service delivery and teaching, with proportionally less time for research. Consequently, capacity building of research assistants and data managers is important, and the establishment of energy-efficient data centres is necessary on the continent.

## Strengths and limitations

This qualitative study supplements the quantitative survey on health researchers conducted in SSA [37]. As such, it makes a valuable contribution to the field of data-intensive research in SSA. It has also led to a more detailed in-depth study on health researchers in South Africa (in preparation for submission).

Although this study has yielded rich data on the challenges faced by researchers in SSA, many Francophone and Lusophone countries are not represented. This is due to the research being conducted by an English-speaking team, the lack of French and Portuguese-speaking researchers in SSA with expertise in data-sharing and the high costs of translation of transcripts from other languages to English. Obtaining 16 IDIs took nine months of concerted effort with challenges related to internet access, lack of response to email invites and connectivity issues, so aiming for a bigger sample size would require several more months. The aim was not to reach saturation per country but to obtain an overall picture of challenges experienced by health researchers from different SSA countries. We have completed an in-depth study in one country—South Africa—to be submitted for review shortly. So, data saturation was reached from a continental perspective in that respondents from different countries expressed similar views by interview 16 but country saturation in the wider SSA region was not reached.

Additionally, this research study was exploratory and descriptive; hence the aim is not to generalise the findings but to provide a nuanced understanding of data sharing and challenges experienced by health researchers. For this reason, our sample size, although small, is sufficient for the type of study conducted. Qualitative research, unlike quantitative research, is valid with a small sample [94].

Some challenges in recruitment can be mitigated by practices that include surveying stakeholders through multiple contacts, personalising communication messages and using prepaid incentives are some suggestions for future improvements. This is an area for further research in different countries. We have expanded the qualitative enquiry in SA in this manner and the same could be done for other countries. Despite the limitations, data obtained after 16 interviews proved to be valuable.

Resources are urgently needed in SSA to improve the quality, value and veracity of health data–as these are ethical imperatives. Strengthening data governance, boundary-setting and standardising data sharing agreements will increase trust among researchers and data donors [8,10,40]. Verdegem (2022) contributes that ‘treating data as capital allows for a more nuanced and detailed understanding of AI capitalism functions’ [2]. The main concern for health researchers is to realize the juncture between AI systems and data sharing as a capital investment that future health research may address [92].

## Conclusion

There is significant discomfort about data sharing in the context of collaborative research amongst health researchers in this sample of respondents from SSA countries resulting in a reluctance to share despite acknowledgement of the scientific benefits of such sharing. While data intensive research and data sharing appear to be increasing researcher vulnerability at an individual level, challenges to data sharing exist at a broader collective level as well. In particular, suboptimal data quality in LMICs as a result of human, structural and financial resource limitations remain a serious threat to seamless sharing globally. It is essential to allay anxieties about the protection of data via robust governance frameworks to ensure that health data sharing is beneficial to all research stakeholders. Health researchers will be optimistic about depositing, retrieving, modifying, moving or sharing stored data on platforms if they know that their scientific currency is safeguarded within a regulated context.

## Supporting information

S1 File Interview guide. (DOCX)
